# Vergleichende Analyse der Refraktions- und Topographieveränderungen nach Augenmuskeloperationen

**DOI:** 10.1007/s00347-021-01456-8

**Published:** 2021-07-08

**Authors:** J. Mehlan, H. Thormählen, M. K. Casagrande, E. S. Lachmann, V. Druchkiv, D. Bittersohl, M. Spitzer, F. Schüttauf

**Affiliations:** Klinik und Poliklinik für Augenheilkunde, Universitätsklinikum Hamburg-Eppendorf, Martinistr. 52, 20246 Hamburg, Deutschland

**Keywords:** Strabismus, Hornhaut, Zylinder, Augenmuskeln, Wundheilung, Strabismus, Cornea, Cylinder, Eye muscle, Wound healing

## Abstract

**Hintergrund:**

Über die Frage, ob nach Augenmuskeloperationen Refraktions- oder Topographieveränderungen zu erwarten sind, besteht weitestgehend Unklarheit.

**Material und Methoden:**

In der vorliegenden Studie wurden prä- sowie postoperativ von 229 Augen objektive Refraktion, Pentacam (Fa. Oculus, Menlo Park, CA, USA) und Visus erhoben und analysiert. Die Untersuchung erfolgte präoperativ, am ersten postoperativen Tag sowie nach 3 Monaten.

**Ergebnisse:**

Nach Operationen an einem sowie 2 geraden Augenmuskeln bestehen signifikante Veränderungen des Astigmatismus (*p* < 0,001), jedoch normalisiert sich dieser nach 3 Monaten wieder auf das präoperative Niveau. Ebenso war dieser Verlauf nach kombinierten Operationen von geraden und schrägen Augenmuskeln zu belegen.

**Schlussfolgerung:**

Es lässt sich daher postulieren, dass eine umfangreiche Aufklärung von Patienten hinsichtlich passagerer Veränderungen des Zylinders insbesondere bei Beteiligung gerader Augenmuskeln unabdingbar ist und postoperativ eine ergänzende Refraktions- und Topographiekontrolle bei mangelnder Visusrehabilitation sinnvoll sein kann.

## Abstract (en)

**Background:**

The question of whether refractive or topographic changes are to be expected after eye muscle surgery is largely unanswered.

**Materials and methods:**

Results of pre- and postoperative objective refraction, Pentacam (Oculus, Menlo Park, CA, USA), and visual acuity tests of 229 eyes are included in the present analysis. The examinations took place preoperatively, on the first postoperative day, and after 3 months.

**Results:**

After surgery on one or two extraocular recti muscle, there are significant changes in the cylinder (*p* < 0.001) on the first postoperative day, although values returned to normal after 3 months. Similar changes also appeared in combined operations of extraocular and oblique muscles.

**Conclusion:**

The authors postulate that extensive patient education with regard to temporary changes in the cylinder is necessary, particularly when the extraocular muscles are involved, and that additional refraction and topography control can be useful postoperatively if visual rehabilitation is inadequate.

Kurvatur und Topographie der Hornhaut werden durch zahlreiche Faktoren beeinflusst. Nachfolgend wirken sich diese auf die Refraktion, insbesondere auf den Astigmatismus, aus.

Muskuläre Einflüsse, wie beispielsweise vom M. orbicularis oculi ausgehend [2], oder die Position der Lider können die Topographie beeinflussen [5], aber auch dellende Maßnahmen der Sklera spielen eine Rolle [6].

Durch die Desinsertion bzw. Reintegration von Ausgenmuskeln im Zuge der Operation und durch die veränderten Auflagepunkte auf der Sklera kann es zu Veränderungen der Refraktion kommen. Ebenso kann der ggf. postoperativ entstehenden Narbenzug die Refraktion verändern.

Es herrscht unter den Fachkollegen nach wie vor keine Einigkeit, ob es durch Augenmuskeloperationen zu einer Veränderung der Refraktion oder der kornealen Topographie kommt. Auch inwiefern diese Änderungen von Dauer sind, ist noch nicht abschließend geklärt [1, 3].

Klar ist, dass es direkt postoperativ durch die aus der Operation resultierende Schwellung und auch durch die medikamentösen Versorgung, v. a. bei Salbenapplikation, zu Visusschwankungen oder auch -reduktionen kommen kann [7].

Mit diesen Untersuchungen wollten wir evaluieren, ob es durch die operative Korrektur der Schielstellung zu Veränderungen von Refraktion, Visus oder kornealer Topographie kommt und ob es Unterschiede hinsichtlich des zu operierenden Muskels gibt.

## Methodik

Im Studienzeitraum von Oktober 2019 bis März 2020 wurden 305 Patienten an den Augenmuskeln operiert.

Ausgenommen wurden von dieser Evaluation mit nichtstrabologischen Bereichen kombinierte Operationen (beispielsweise mit lidchirurgischen Eingriffen) oder auch Reoperationen der Augen (im Zeitraum von 3 Jahren rückwirkend zum Operationszeitpunkt).

Alle Eingriffe wurden von 2 erfahrenen Operateuren in gleicher Technik durchgeführt. So sollten möglichst viele Bias durch z. B. stärkere Schwellung bei kurzfristigen Reoperationen ausgeschlossen werden.

Es erfüllten 188 Patienten mit insgesamt 229 zu operierenden Augen die Einschlusskriterien und willigten ein, an der prospektiven Studie teilzunehmen.

Das Follow-up absolvierten 35 Patienten vollständig.

Leider mussten aufgrund der derzeitigen Pandemiesituation und den damit verbundenen Reduzierungen der Patientenzahlen ein großer Teil der Kontrollen nach 3 Monaten abgesagt werden, da diese nicht als medizinisch essenziell einzustufen waren.

Aus diesem Grund sind zu dem Kontrollzeitpunkt nach 3 Monaten in den jeweiligen Gruppen entsprechend weniger Ergebnisse verzeichnet.

Das Vorhaben wurde durch die örtliche Ethikkommission beraten und genehmigt.

### Ablauf

Nach vorangegangener Information und Aufklärung wurden die Patienten präoperativ sowie am ersten postoperativen Tag und nach 3 Monaten mittels objektiver Refraktion (NIDEK-ARK-560A [NIDEK CO., LTD, Japan]) und Pentacam (Pentacam®, Fa. Oculus, Menlo Park, CA, USA*) *untersucht. Zusätzlich erfolgte zu jedem der 3 Zeitpunkte eine Visusprüfung.

Die Gruppierung erfolgte entsprechend der durchgeführten Operation (Gruppe 1: Operation an einem geraden Augenmuskel, Gruppe 2: Operation an 2 geraden Augenmuskeln, Gruppe 3: Operation an einem schrägen Augenmuskel, Gruppe 4: kombinierte Operation aus geraden und schrägen Augenmuskeln, Gruppe 5: Operation beider schräger Augenmuskeln).

### Operation

Alle Operationen erfolgten nach dem gleichen Prinzip von 2 erfahrenen Operateuren.

Nach dem Team-Time-out erfolgten die Desinfektion und das Abdecken des Operationsgebiets und Anschlingen des Bulbus mit Mersilenefäden.

Nach der Eröffnung der Bindehaut wurden die Muskelränder durch vorsichtiges Spreizen mit dem Scherchen dargestellt, das Tenongewebe stumpf vom Muskel entfernt.

Die Bindehauteröffnung erfolgte bei allen Operationen sehr schonend und bei den geraden Augenmuskeln oder bei der Operation von geraden und schrägen Augenmuskeln mit einem Türflügelschnitt. Die Operation eines reinen schrägen Augenmuskels erfolgte über eine radiäre Bindehauteröffnung.

In allen Fällen wurde minimal-invasiv gearbeitet, und der Wundverschluss erfolgte vom Limbus ausgehend.

In unserem Patientengut wurden lediglich Rücklagerungen oder Resektionen der geraden Muskeln sowie Rücklagerungen oder Faltungen der schrägen Augenmuskeln vorgenommen.

Für die Rücklagerungen wurden 2 Ecknähte am Muskel vorgelegt und mithilfe dieser der Muskel nach dem Abtrennen und Abmessen der Strecke mit dem Zirkel exakt wieder an der Sklera fixiert.

Im Fall einer Resektion wurde die Strecke abgemessen, eine Muskelklemme positioniert und der Muskel nach dem Abtrennnen mittels U‑Naht nach Harms in der Muskelansatzleiste wieder fixiert. Überstehende Muskelteile wurden entfernt.

Die Faltungen der schrägen Augenmuskeln erfolgten nach Aufnehmen auf den Schielhaken mithilfe zweier fixierender Vicrylnähte.

Für die Rücklagerungen bzw. Transpositionen wurden im Bereich der Muskelansatzleiste Ecknähte vorgelegt, der Muskel abgetrennt und nach Abmessen der entsprechenden Strecke skleral fixiert.

### Statistik

Um die Änderung der Messungen bei jeder Variablen zu analysieren, wurde die gemischte Regression geschätzt. Der Vorteil der gemischten Regression besteht u. a. darin, dass alle Messungen aller Augen verwendet werden können und nicht nur die Messungen von den Augen mit kompletten Daten (wie in klassischer Varianzanalyse [englisch analysis of variance, abgekürzt ANOVA]). Insbesondere ist es in unserem Fall sehr nützlich, weil wir eine starke Reduktion der Daten zu 3 Monaten nach der Operation beobachten.

Die gemischte Regression mit dem Auge-Effekt als Zufallseffekt macht 2 Annahmen [4].

Annahme 1 – Die Fehler innerhalb der Gruppen sind unabhängig, identisch normalverteilt, mit mittlerer Varianz gleich null, und sie sind unabhängig von Zufallseffekten.

Annahme 2 – Die Zufallseffekte sind normalverteilt, mit mittlerer Varianz gleich null.

Diese Annahmen wurden mittels der Beurteilung der Residuen-Grafiken geprüft. Im Fall der Probleme wurde alternativ eine robuste gemischte Regression geschätzt, wo die ausreißenden Fälle nach Huber-Methode gewichtet werden.

Die Refraktion wurde in Power-Vektoren [9] umgerechnet, und die Änderung der 3 Komponente (sphärisches Äquivalent, J0 und J45) wurde wiederum mittels gemischter Regression statistisch geprüft.

Alle Berechnungen wurden mit R Core Team (2019) durchgeführt.

Zu beachten ist, dass unser Studiendesign die Unterschiede zwischen gebundenen Stichproben analysiert. Hierbei ist es möglich, dass sich die Konfidenzintervalle der geschätzten Mittelwerte zu den zu vergleichenden Zeitpunkten überlappen können, während der Unterschied signifikant ist.

## Ergebnisse

Insgesamt konnten 229 Augen in unsere Auswertung eingeschlossen werden.

Es haben lediglich 35 Patienten das Follow-up vollständig absolvieren können. Die verbleibenden Datensätze haben wir jedoch, unter jeweiliger Berücksichtigung der Stichprobenzahl zu den jeweiligen Zeitpunkten, trotzdem in die Auswertung einfließen lassen. Im Rahmen der COVID-19-Pandemie war es uns über einen großen Zeitraum der Studie nicht möglich, diese Patienten erneut einzubestellen. Die Entscheidung erfolgte im Zuge einer sorgfältigen Risikoabwägung für die betreffenden Patienten und auch das Personal der Abteilung.

### I Operation an einem geraden Augenmuskel

In diese Gruppe konnten 80 Augen eingeschlossen werden, an denen eine Augenmuskeloperation im Sinne einer Ein-Muskel-Chirurgie durchgeführt wurde. Das vollständige Follow-up lag, aufgrund der geschilderten Situation, bei 10 untersuchten Augen vor.

**Figure 1:**
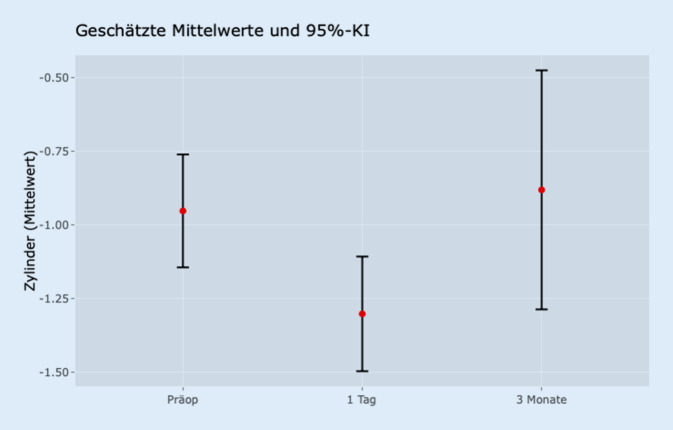

In dieser Patientengruppe zeigte sich keine signifikante Veränderung der Sphäre zwischen den jeweiligen Zeitpunkten. Jedoch änderte sich der Zylinder (Abb. 1) signifikant beim Vergleich der präoperativen Daten mit den Werten am ersten postoperativen Tag (*p* < 0,001). Nach 3 Monaten bewegte sich dieser Wert wieder auf das ursprüngliche Niveau, sodass kein signifikanter Unterschied mehr zu den präoperativen Daten bestand (*p* = 0,932).

Ebenso zeigte sich eine signifikante Reduktion des Visus zwischen der präoperativen und der ersten postoperativen Messung (*p* < 0,001). Im Vergleich zur Messung nach 3 Monaten war der Visus jedoch wieder auf das ursprüngliche Niveau angestiegen (*p* = 0,836).

### II Operation an 2 geraden Augenmuskeln

In diese Gruppe konnten 65 Operationen eingeschlossen werden, jedoch konnten nur 6 Patienten das Follow-up vollständig absolvieren.

**Figure 2:**
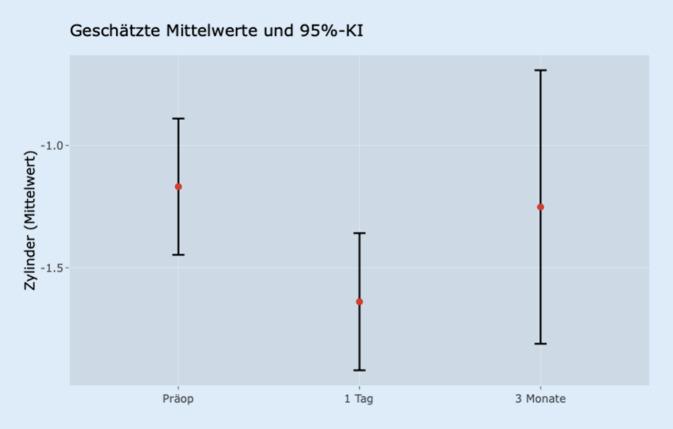

Bei der Analyse der Sphäre gab es keine signifikanten Veränderungen zwischen den 3 Zeitpunkten. Zur Analyse des Zylinders ist zunächst zu konstatieren, dass es im Vergleich von der präoperativen Kontrolle zur ersten postoperativen Kontrolle eine signifikante Veränderung des Zylinders gab (*p* < 0,001). Der Unterschied hingegen zur Kontrolle nach 3 Monaten war statistisch nicht signifikant (*p* = 0,947). Diese Entwicklung ist in Abb. 2 dargestellt.

Auch in dieser Gruppe war die Reduktion des Visus zwischen der präoperativen Testung im Vergleich zum Visustest nach der Op. statistisch signifikant (*p* = 0,002).

Nach 3 Monaten war dieser Wert jedoch wieder auf dem Ursprungsniveau und der Unterschied nicht mehr als signifikant zu belegen (*p* = 0,978).

Die zentrale Pachymetrie, welche mit der Pentacam ermittelt wurde, wies von der präoperativen Messung zur Messung am ersten postoperativen Tag eine signifikante Erhöhung auf (*p* < 0,001), und nach 3 Monaten hatte sich dieser Wert auf das Ausgangsniveau stabilisiert (*p* = 0,955).

### III Operation an einem schrägen Augenmuskel

Im Studienzeitraum erhielten 41 Augen lediglich eine Korrektur an einem schrägen Augenmuskel. Von diesen absolvierten 10 das Follow-up vollständig.

**Figure 3:**
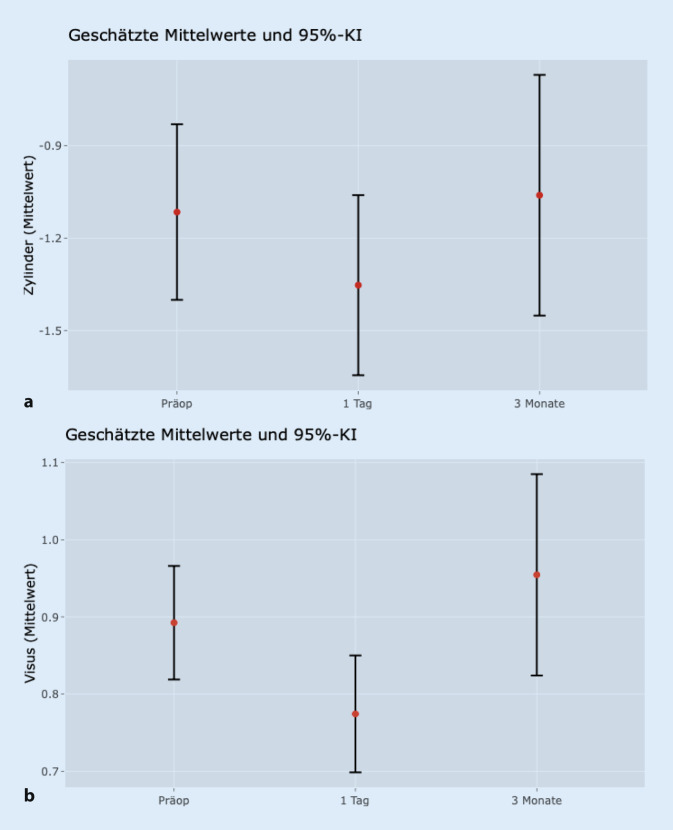

In dieser Patientengruppe zeigte sich keine signifikante Veränderung der Sphäre zwischen den jeweiligen Zeitpunkten. Auch der Zylinder wies keine statistisch signifikanten Veränderungen zwischen den jeweiligen Zeitpunkten auf (*p* > 0,05) (Abb. 3a).

Ebenso zeigte sich eine signifikante Reduktion des Visus zwischen der präoperativen und der ersten postoperativen Messung (*p* < 0,013). Im Vergleich zur Messung nach 3 Monaten ist der Visus jedoch wieder auf das ursprüngliche Niveau angestiegen (*p* = 0,631). Dies ist in Abb. 3b im Boxplot aufgetragen.

Auch die zentrale Pachymetrie konnte hier keine signifikanten Veränderungen durch die operative Prozedur belegen.

### IV Kombinierte Operation gerader und schräger Augenmuskeln

In diese Gruppe konnten 38 Operationen eingeschlossen werden, jedoch konnte nur bei 8 untersuchten Augen das Follow-up vollständig absolviert werden.

**Figure 4:**
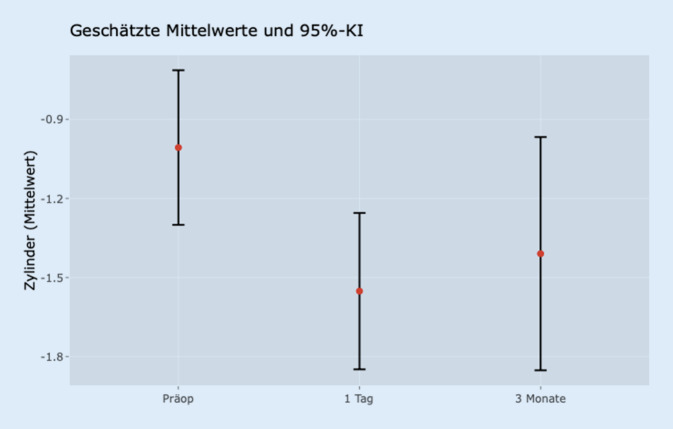

Bei der Analyse der Sphäre gab es keine signifikanten Veränderungen zwischen den 3 Zeitpunkten. Zur Analyse des Zylinders ist zunächst zu konstatieren, dass es im Vergleich von der präoperativen Kontrolle zur ersten postoperativen Kontrolle eine signifikante Veränderung des Zylinders gab (*p* < 0,001). Der Unterschied hingegen zur Kontrolle nach 3 Monaten war statistisch nicht signifikant (*p* = 0,118). In Abb. 4 sind die Daten im zeitlichen Verlauf dargestellt.

Auch in dieser Gruppe war die Reduktion des Visus zwischen der präoperativen Testung im Vergleich zum Visustest nach der Op. statistisch signifikant (*p* < 0,001).

Nach 3 Monaten waren die Differenzen nicht mehr als signifikant zu belegen (*p* = 0,3).

Die zentrale Pachymetrie, welche mit der Pentacam ermittelt wurde, wies von der präoperativen Messung zur Messung am ersten postoperativen Tag eine signifikante Erhöhung auf (*p* < 0,001), und nach 3 Monaten hatte sich dieser Wert auf das Ausgangsniveau stabilisiert (*p* = 0,651).

### V Operation an beiden schrägen Augenmuskeln

In diese Gruppe konnten 5 Augen eingeschlossen werden. Lediglich ein Patient konnte aufgrund der geschilderten Situation auch die Kontrolle nach 3 Monaten absolvieren.

**Figure 5:**
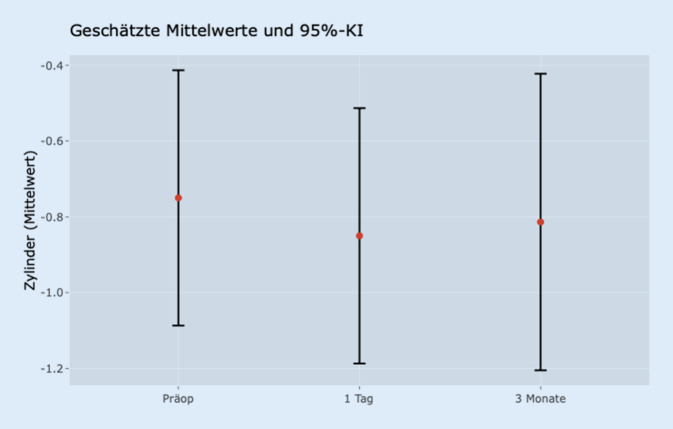

In dieser Patientengruppe zeigte sich keine signifikante Veränderung der Sphäre zwischen den jeweiligen Zeitpunkten. Auch der Zylinder (Abb. 5) wies keine signifikanten Veränderungen beim Vergleich der präoperativen Daten mit den Werten zum ersten postoperativen Tag oder zur Kontrolle nach 3 Monaten auf.

Der Visus ist statistisch nicht signifikant verändert.

Lediglich die Pachymetrie zeigte beim Vergleich der Werte zwischen der präoperativen Kontrolle und dem ersten postoperativen Tag eine signifikante Veränderung (*p* = 0,035) und normalisierte sich im Verlauf wieder, sodass zur Kontrolle nach 3 Monaten im Vergleich zum Ausgangsniveau keine signifikante Veränderung mehr zu belegen war (*p* = 0,116).

## Diskussion

Ziel der vorgelegten Arbeit ist die Klärung der Fragestellung, ob und inwiefern Operationen an den extraokularen Augenmuskeln einen Effekt auf die Refraktion bzw. die Hornhauttopographie haben.

Es existieren einige Voruntersuchungen, von denen wir einige im Folgenden aufgreifen und zur Diskussion stellen wollen.

Der große Unterschied im Vergleich zu unserer Studie beruht darin, dass wir die einzelnen Operationsindikationen vergleichend und im selben Setting einander gegenüberstellen konnten und hierbei auch die in der klinischen Praxis häufige kombinierte Operation von geraden und schrägen Augenmuskeln untersuchen konnten.

Leshno et al. führten 2017 eine Studie mit 31 Patienten durch, in welcher die refraktiven Veränderungen nach Operationen an den geraden horizontalen Augenmuskeln untersucht wurden. Es zeigte sich kein signifikanter Unterschied hinsichtlich der Refraktionsänderung zwischen der Operation des äußeren (M. rectus lateralis) und des inneren Augenmuskels (M. rectus medialis) [1]. In dieser Studie wurde das nicht operierte Auge mit dem jeweils operierten verglichen, und als klinisch signifikant wurden Veränderungen über 0,75 dpt bewertet. Sphäre und Zylinder veränderten sich häufiger klinisch signifikant in den operierten Augen als in den unoperierten (Sphäre: 48 vs. 10 %; Zylinder 32 vs. 10 %) [1].

Nardi et al. untersuchten 1997, inwiefern sich die korneale Topographie nach Operationen an den geraden horizontalen Augenmuskeln (*n* = 36) verändert [3].

Sie konstatierten, dass der korneale Astigmatismus am ersten Tag signifikant verändert war. Vor allem in der optischen Zone waren die Veränderungen nach einem Monat wieder rückläufig [3].

Vergleicht man die Daten mit unseren Ergebnissen, so konnten wir, aufgrund unseres Studiendesigns, die signifikante Veränderung des Zylinders sowohl für Operationen an einem als auch an 2 geraden Augenmuskeln nachweisen und zudem noch belegen, dass, auch wenn im Rahmen der Chirurgie an einem schrägen äußeren Augenmuskel ein gerader Muskel mit operiert wird, sich der Zylinder signifikant – zumindest temporär verändert.

Wir haben im Gegensatz zur Arbeitsgruppe um Nardi et al. eine postoperative Nachbeobachtungzeit von 3 Monaten gewählt, um den Effekt nach abgeschlossener Wundheilung abbilden zu können.

Snir et al. fanden 1989 eine leichte Tendenz zur postoperativen Myopie in der Population von 23 Kindern, welche an den horizontalen Augenmuskeln operiert wurden [8].

Dies lässt sich mit unseren Analysen nicht abbilden. Es besteht demnach auch in der großen Patientengruppe kein Hinweis auf eine signifikante Veränderung der Sphäre.

In einer Studie der Arbeitsgruppe um Schworm wurden 77 Patienten 1996 vergleichend untersucht. Diese Zahl Patienten umfasste sowohl die Operation gerader als auch einiger schräger Augenmuskeln (Obl.-inferior-Korrekturen *n* = 10; andere, z. B. Obl.-superior-Operationen *n* = 6).

Im Unterschied zu dieser angeführten Arbeit wurden unsere Patienten alle im gleichen operativen Setting (z. B. auch identische Narkoseform) versorgt, um Bias wie hier beispielsweise durch Operation eines Teils der Patienten in Retrobulbäranästhesie auszuschließen.

Des Weiteren zeigt unsere klinische Praxis, dass es auch gehäuft Indikationen mit einer Kombination aus schrägen und geraden Augenmuskeln gibt, sodass wir diese Gruppe zusätzlich betrachten wollten.

Nichtsdestotrotz stimmen wir mit den Kernaussagen der Autoren überein. Diese konstatieren ebenso, dass es sich meist um eine vorübergehende Refraktionsänderung handelt. Im Fall persistierender Visusbeeinträchtigungen sollte auch eine längerfristig anhaltende Refraktionsänderung in Erwägung gezogen werden [7].

Als deutlichste Limitation unserer Studie ist die Reduktion der Patientenzahl zur Kontrolle nach 3 Monaten zu sehen. Hier war es uns aufgrund der derzeitigen Situation und dem Erfordernis, die Patientenkonsultationen auf ein notwendiges Minimum zu begrenzen, nicht möglich gewesen, einen vollständigen Datensatz zu erheben.

Wir planen jedoch, diesen Zeitpunkt im Sinne einer Folgestudie mit weiteren Patienten erneut zu betrachten. Des Weiteren muss erwähnt werden, dass die Aussagen lediglich für die hier durchgeführten Prozeduren anzuwenden sind. Des Weiteren ist anzumerken, dass zum Kontrollzeitpunkt am ersten postoperativen Tag das Vorhandensein einer Bindehautschwellung und deren Ausmaß einen Einfluss auf die Ergebnisse hat. Wir haben uns dennoch für diesen Kontrollzeitpunkt entschieden, um den Patienten, welche zumeist große Anfahrtswege haben, eine weitere Anfahrt zu ersparen.

Die Vorteile unserer Studie liegen unseres Erachtens darin, dass verschiedene Operationsverfahren bzw. Indikationen im gleichen Setting betreut und verglichen werden können. Hierfür konnten wir jeweils eine ausreichend große Patientengruppe rekrutieren.

So konnten wir aus unserer Sicht trotz der Erschwernisse der derzeitigen Pandemiesituation die eingangs gestellte Frage hinreichend beantworten.

So lässt sich anhand unserer Daten konstatieren, dass die Augenmuskeloperation nachweislich einen Einfluss auf die Hornhauttopographie und die Refraktion hat. Jedoch stellt sich dieser Effekt bislang als vorübergehend dar. Nach 3 Monaten konnten wir keinen signifikanten Unterschied zum Ausgangsniveau mehr feststellen.

Zudem empfiehlt es sich unserer Ansicht nach, die vorgelegten Daten insofern für die klinische Praxis zu berücksichtigen, als bei mangelnder Visusrehabilitation nach einer Augenmuskeloperation die Refraktion, im Fall der Kinder auch skiaskopisch, und ggf. die Hornhauttopografie überprüft werden sollten.

## Fazit für die Praxis

- Vor einer Augenmuskeloperation sollte auf die Möglichkeit einer temporären Änderung des Astigmatismus hingewiesen werden, v. a. wenn es sich um eine Chirurgie der geraden extraokularen Muskulatur handelt.
- Postoperativ sollten bei mangelnder Visusrehabilitation die Refraktion (je nach Alter des Patienten ggf. skiaskopisch) und die Topographie überprüft werden.
- Gerade im Kindesalter sollte ein korrekter Refraktionsausausgleich aufgrund des amblyogenen Risikos sorgfältig überwacht werden.
